# Retraction Note: Calcium-dependent transcriptional changes in human pancreatic islet cells reveal functional diversity in islet cell subtypes

**DOI:** 10.1007/s00125-023-05983-8

**Published:** 2023-08-04

**Authors:** Ji Soo Yoon, Shugo Sasaki, Jane Velghe, Michelle Y. Y. Lee, Helena Winata, Cuilan Nian, Francis C. Lynn

**Affiliations:** 1 Diabetes Research Group, BC Children’s Hospital Research Institute, Vancouver, BC Canada; 2 CELL Graduate Program, Faculty of Medicine, University of British Columbia, Vancouver, BC Canada; 3 Department of Surgery, Faculty of Medicine, University of British Columbia, Vancouver, BC Canada; 4 School of Biomedical Engineering, University of British Columbia, Vancouver, BC Canada

**Retraction Note: Diabetologia (2022) 65:1519-1533**

10.1007/s00125-022-05718-1

The authors have retracted this article following the discovery of a data processing error that impacts the results. Specifically, the number of cells sequenced and analyzed was significantly lower than reported in the article. The authors have reanalyzed the data and concluded that, while the overall conclusions have not changed, many of the details have. The nature of this error makes correction unfeasible.

The authors thank colleagues for bringing this error to their attention and apologize to the community.

All authors agree with this retraction.

